# The Gift of Expression

**Published:** 1920-09-18

**Authors:** 


### THE GIFT OF EXPRESSION.

**BY A MATRON.**

Before war devastated the face of Europe the habitual *sang-froid* of the English people had become a by-word amongst Continental nations, the inherent reserve of the individual was regarded as silently expressed contempt for manners and customs not essentially British. After nearly five years of comradeship, cemented by suffering and death, the volatile Latin rubbed his eyes and awakened to the fact that in reality the Englishman's curious aloofness was due to innate shyness and an exaggerated fear of ridicule. Individually and collectively, as a nation, we distrust all emotional expression.

If modern education has this stultifying effect upon the national imagination, what must be the further effect of the curricula of the nurse training-schools upon the minds of the probationers who spend three or four years beneath their ægis?

The answer is to be found in the frequent lethargy of the rank and file of the nursing services. They are asked, implored, cajoled, by those amongst them who have the foresight and the imagination to see the dire necessity for co-ordination and reconstruction, to express their wishes, and to shape their thoughts for definite action. Their only answer is silence, no flicker of enthusiasm, no flash of vivid understanding; it is as if they had no interest in the questions of the present day, as if a gate, locked and barred, stood between them and legitimate development.

Can it be that three or four years of training has so devitalised them mentally that it has completed what the process of educational repression had commenced, and that whatsoever they had retained of individuality has become merged into communal thought? If this is so, it is a terrible charge to bring against a system that has been and still is the model from which other nations have drawn their inspirations and their ideals.

The science of nursing still rests upon the bed-rock of conventual vocation, but the modern nurse has to contend with difficulties and dangers of which the *réligieuse* was completely ignorant. Not only has she to consider the well-being of her patients, she has also to consider the matter of her own maintenance and the provision for her future, when her economic life is done. Nursing is a science which is continually advancing in knowledge; the nurse straight from her training-school may throw aside her text-books and feel her days of study are ended, but at the conclusion of six months' rest she is obliged to attend post-graduate lectures and to take an intelligent interest in the ever-changing social economic problems of the day or to become of no account in the web of national endeavour.

Are the nurse training-schools of to-day fitting the women who are undergoing their curricula for the wide heritage which awaits them?

If we are to have an efficient Health Service we must send into its ranks broad-minded women of well-trained intelligence, not machines deprived of the power of further mental development; we must replace conventional traditions with high professional ideals; we must instil into the minds of nurse-probationers the value of individuality and the need for self-expression.

If the Nursing Services of the future are to uphold the traditions of the past we must widen our curricula; we must teach the value of debate, the possibility of discussion, and the responsibility for individual opinion.

Every woman who enters the Nursing Services owes a duty to her patients, to herself, and to her fellow-workers; she is under a series of obligations which she is morally compelled to discharge, and by neglecting the one she is also neglecting the others. It is essential to the welfare of the people of these islands that the Nursing Services should be skilful, efficient, well-trained, and well-disciplined, competent to settle their internal differences, and to draw up a clear and lucid statement of their vital needs for the consideration of the public; but this can only be attained by mental intercourse and generous discussion as opposed to the lethargic indifference and apathetic silence which, unhappily, is so prevalent to-day.

